# Evaluation of the Incidence of Malignancy in Sjögren's Syndrome: A Single-Center Study From Turkey

**DOI:** 10.7759/cureus.81219

**Published:** 2025-03-26

**Authors:** Tugba Kahraman Denizhan, Emel Oguz Kokoglu, Melih Kızıltepe, Celil B Cengiz, Ismail H Tuncez, Abdurrahman S Senel

**Affiliations:** 1 Division of Rheumatology, Department of Internal Medicine, Erciyes University Faculty of Medicine, Kayseri, TUR; 2 Department of Public Health, Konya Provincial Health Directorate, Konya, TUR

**Keywords:** cancer, incidence, lymphoma, malignancy, sjögren's syndrome

## Abstract

Objective: Sjögren’s syndrome (SS) is known to be associated with an elevated cancer incidence. This study aimed to ascertain SS patients’ cancer incidence rates and risk variables at a single center in Turkey.

Methods: The clinical data of SS patients were analyzed retrospectively. Cancer incidences for the overall population were obtained. The standardized incidence rates (SIRs) of hematological and solid cancers were calculated in comparison with the overall population.

Results: The study included 303 SS patients, of whom 27 (8.9%) were diagnosed with cancer. Twenty-one solid (6.9%) and six (2%) hematologic cancers were identified. The SIR for all cancers was 2.25 (95% confidence interval (CI) 1.513-3.228). The SIR for hematologic cancers was 7.22 (95% CI 2.93-5.04), while the SIR for non-Hodgkin lymphoma (NHL), the most prevalent malignancy, was 11.76 (95% CI 3.73-28.38). The most prevalent malignancies were papillary thyroid and breast cancers, with a SIR of 3.04 (95% CI 1.937-4.58) for solid cancers. The SIR for both papillary thyroid and breast cancers was 8.43 (95% CI 3.689-16.68) and 1.85 (95% CI 0.687-4.105), respectively. A higher risk has also been linked to the presence of lymphopenia.

Conclusion: SS patients exhibit an increased risk of both solid and hematologic malignancies compared to the total population. This study reveals an increased risk of NHL and thyroid cancer in the Turkish population.

## Introduction

Sjögren’s syndrome (SS) is a chronic autoimmune and inflammatory disorder characterized by lymphoplasmacytic infiltration, primarily affecting the lacrimal, salivary, and other exocrine glands [1]. While SS typically targets exocrine tissues, it can also affect multiple organ systems. It manifests as primary SS (pSS) when occurring independently or secondary SS (sSS) when associated with other diseases. Beyond common symptoms like dry eyes and mouth, systemic involvement arises through gland dysfunction, autoimmune epithelitis, organ-specific autoimmunity, or immune complex-related complications. Approximately 75% of SS patients experience extraglandular symptoms, ranging from mild joint pain to severe, life-threatening complications [2].

The pathogenesis of SS involves abnormal immune responses primarily driven by T and B lymphocytes. It progresses in three phases: (1) a trigger phase, influenced by genetic susceptibility, hormonal, and epigenetic factors; (2) dysfunctional epithelial activity in exocrine glands; and (3) a chronic inflammatory phase marked by lymphocytic infiltration, increased B-cell activity, and autoantibody production [3].

Several studies indicate increased mortality in pSS patients compared to the general population. Mortality is especially elevated in patients with interstitial lung disease (ILD), with worse outcomes observed in male patients and those of advanced age. Systemic organ involvement, lymphoma, and the use of corticosteroids and immunosuppressive drugs are also associated with a higher risk of mortality [4]. pSS patients are 10-44 times more likely to develop lymphoma compared to healthy individuals, with mucosa-associated lymphoid tissue (MALT) lymphoma, marginal zone lymphoma, and diffuse large B-cell lymphoma accounting for 90% of cases [5].

Additionally, pSS patients show a higher incidence of lung cancer, breast cancer, and thyroid cancer, as well as non-melanoma skin cancer, compared to the general population [6-8]. Although certain risk factors, such as neutropenia, splenomegaly, and cryoglobulinemia, are linked to lymphoma development, the risk factors for solid tumors remain unclear [9]. Our study aims to investigate the incidence of malignancies and associated risk factors in SS patients within a single-center Turkish cohort.

## Materials and methods

This retrospective study investigated the incidence and types of cancer in SS patients treated at Erciyes University Faculty of Medicine, Rheumatology Clinic, between January 2011 and February 2023. The study included pSS and sSS patients who met the 2016 American College of Rheumatology/European League Against Rheumatism (ACR-EULAR) classification criteria and excluded those under 18 years of age, those not meeting the criteria, and those diagnosed with malignancy before SS [10].

After ethics committee approval, patient data with the diagnosis code M35 were retrieved from the hospital information system. Anamnesis records, pathology results, and clinical files were reviewed to collect information on disease duration, time to cancer diagnosis, and cancer type. Data on age, sex, smoking history, medications, comorbidities, acute phase reactants, and antibody positivity were also collected.

Clinical variables included disease duration, sex, age, and the presence of dry mouth/eyes, joint symptoms, pulmonary involvement, and Raynaud’s phenomenon. Laboratory data such as complete blood count, antinuclear antibodies (ANAs), anti-Ro/La antibodies, rheumatoid factor (RF), C3/C4 complement levels, biochemical tests, erythrocyte sedimentation rate (ESR), immunoglobulin levels, and C-reactive protein (CRP) were evaluated using electronic medical records. Reference ranges defined anemia as hemoglobin (Hb) < 12 g/dL in women and <14 g/dL in men, leukopenia as WBC < 4.0 × 10⁹/L, and thrombocytopenia as platelets < 150 × 10⁹/L. ANA positivity was defined as a titer > 1/100, and RF positivity was measured via nephelometry. The cut-off values for C3 and C4 were 80 and 10 mg/dL, respectively. Elevated ESR and CRP were defined as >20 mm/h and >0.5 mg/dL, respectively.

Data analysis was performed using IBM SPSS 23.0 (IBM Corp., Armonk, New York, US). Descriptive statistics were presented as median (first to third quartile) and percentages. The Kolmogorov-Smirnov test assessed data normality, while the Mann-Whitney U test was used for continuous variables. Categorical variables were analyzed with the Chi-squared test. Logistic regression analysis identified independent predictors of malignancy in the multivariate analysis. Statistical significance was defined as p < 0.05.

This study was approved by the Erciyes University Hospital Ethics Committee and conducted according to the Helsinki Declaration. The publicly available GLOBOCAN 2022 [11] database was used to access cancer data pertaining to the Turkish population. Follow-up person-years for SS patients were determined from the date of diagnosis to the last visit. Standardized incidence rates (SIRs) were calculated as the ratio of observed to expected cancers in SS patients, stratified by age. The 95% confidence intervals (CIs) of the SIR were also calculated. In this study group, age-specific (over 25 years) SIRs were calculated (there were no patients under 25 years). The cancer incidence for the elderly Turkish population was reported to be 415/100,000. The SIRs by sex could not be calculated in the study, as there were no male cancer patients.

## Results

The study included patients with complete data and who underwent regular follow-ups at Erciyes University Faculty of Medicine, Internal Medicine Rheumatology Outpatient Clinic, between January 2011 and February 2023 and excluded patients with insufficient data. Of the 303 patients with SS, 55 were diagnosed with sSS. Additionally, most sSS patients in our sample were also diagnosed with rheumatoid arthritis (RA) (n = 34). This was followed by systemic lupus erythematosus (SLE) and SS (n = 12) and systemic sclerosis (SSc) and SS (n = 5).

Across all patient groups, the median age was 54 years, the median disease duration was five years, and the number of female patients was 288, accounting for 95% of all patients. Tables 1, 2 display other clinical and laboratory data regarding the patients.

**Table 1:** Comparison of demographic and pathological characteristics of SS patients with and without cancer SS: Sjögren’s syndrome *Independent samples t test

	All patients (n: 303)	Without malignancy (n: 276)	With malignancy (n: 27)	p
Age (years) (median/perc 25-75)*	54 (47-62)	54 (47-62)	58 (51-63)	0.110
Disease duration (years) (median/perc 25-75)*	5 (2-8)	5 (2-8)	5 (3-9)	0.623
Focus score (median/perc 25-75)*	1 (0-2)	1 (0-1.875)	1 (0-2)	0.651

**Table 2:** Comparison of clinical and laboratory characteristics of patients with SS with and without cancer *Independent samples t test **Chi-squared tests SD: standard deviation; ANA: antinuclear antibody; RF: rheumatoid factor; ESR: erythrocyte sedimentation rate; CRP: C-reactive protein; HCQ: hydroxychloroquine; CS: corticosteroids; IS: immunosuppressive (example: conventional synthetic disease-modifying antirheumatic drugs, anti-tumor necrosis factor, azathioprine, mycophenolate mofetil, cyclophosphamide, Janus kinase inhibitors, abatacept, and rituximab); SS: Sjögren’s syndrome

	All patients (n: 303)		Without malignancy (n: 276)		With malignancy (n: 27)		p
	n	%	n	%	n	%	
Women**	288	95	261	96.4	27	100	0.214
Dry mouth**	198	63.5	179	64.9	19	7.0.4	0.565
Dry eyes**	277	91.4	252	91.3	25	92.6	0.820
ANA (≥1/100 dilution)*	271	89.4	246	89.8	23	85.2	0.930
Anti-Ro(+)**	188	62	171	62	17	63	0.995
Anti-La(+)**	44	14.5	43	15.6	1	3.7	0.372
RF (+)**	85	28.1	80	29	5	18.5	0.248
Anemia**	61	20.1	61	22.1	6	22.2	0.777
Leukopenia**	67	22.1	61	22.1	6	22.2	0.988
Lymphocytopenia**	78	25.7	76	27.5	6	22.2	0.022
Thrombocytopenia**	8	2.6	7	2.5	1	3.7	0.718
Low C3/C4**	26	8.6	23	8.3	3	11.4	0.623
Arthralgia and/or arthritis**	222	73.3	203	73.6	19	70.4	0.722
Raynaud’s phenomenon**	16	5.3	15	5.4	1	3.7	0.701
Elevated ESR**	114	37.6	104	37.7	10	37	0.947
Elevated CRP (n/%)**	104	34.3	94	34.1	10	37	0.756
Use of HCQ**	287	94.7	263	95.3	24	88.9	0.156
Use of CS**	111	36.6	104	37.7	7	25.9	0.226
At least one IS**	86	28.4	81	29.3	5	18.5	0.234

Hydroxychloroquine was administered to 94.7% of patients, and the proportion of patients receiving steroids was 36.6%. Conventional synthetic disease-modifying antirheumatic drugs other than hydroxychloroquine were administered to 15.8% of patients, while 28.4% of patients received immunosuppressive therapy. Other treatments received by the patients are detailed in Table 2.

Using the imaging reports, 27 patients were diagnosed with malignancy. Of these patients, 23 (7.6%) had pSS, while four (1.3%) had sSS; all patients were female. While hematologic malignancy was not observed in sSS patients, 17 pSS patients exhibited solid and six patients demonstrated hematologic malignancy. Papillary thyroid cancer (2.3%) and breast cancer (1.9%) were the most prevalent solid malignancies. The most common hematologic malignancy was non-Hodgkin lymphoma (NHL) (1.3%). Other most common malignancies detected in the patients are depicted in Table 3.

**Table 3:** The distribution of types of cancer in patients with SS BCC: basal cell carcinoma; NHL: non-Hodgkin lymphoma; MDS: myelodysplastic syndrome; MF: mycosis fungoides; SS: Sjögren’s syndrome; pSS: primary Sjögren’s syndrome; sSS: secondary Sjögren’s syndrome

	All patients (n: 27)			
	pSS	sSS	Women	Men
All malignancies (n)	23 (7.6%)	4 (1.3%)	27	-
Solid malignancies (n)	17 (5.6%)	4 (1.3%)	21	-
Papillary thyroid cancer	7 (2.3%)	-	1	-
Breast cancer (ductal)	2	-	2	-
Breast cancer (papillary)	3	-	3	-
BCC	2	-	2	-
Endometrium cancer	1	-	1	-
Cervical cancer	1	-	1	-
Lung cancer	-	1	1	-
Colon cancer (adenocarcinoma)	-	1	1	-
Malignant meningioma	-	1	1	-
Myxoid tumors	1	-	1	-
Hemangioendothelioma	-	1	1	-
Hematologic malignancies	6 (2%)			-
NHL	4	-	4	-
MDS	1	-	1	-
MF	1	-	1	-

Research has not identified any relationship between pSS and sSS and malignancy (p = 0.221). There was also no significant association between ILD and malignancy (p = 0.656). No significant relationship was discovered between the malignancy and the treatments administered to the patients (p = 0.402 for anti-tumor necrosis factor, p = 0.820 for azathioprine, p = 0.943 for mycophenolate mofetil, p = 0.470 for cyclophosphamide, p = 0.586 for Janus kinase inhibitors, p = 0.410 for abatacept, and p = 0.211 for rituximab).

Patients with and without cancer were compared for clinical characteristics and laboratory findings. No significant difference was detected in terms of age and disease duration. The laboratory results were generally comparable; however, the non-cancer patient group exhibited a substantial rate of lymphopenia (27.5% vs. 22.5%, p = 0.022).

Table 4 provides the cancer incidence for Turkey and this cohort. Incidences for all cancers as well as the most prevalent solid cancers and hematologic cancers are expressed as 100,000 patient-years in Table 4. SIR was calculated with the observed number of patients, the expected number of patients, and a 95% CI. The most prevalent cancer types were calculated, and the SIR was determined for all cancers, solid cancers, and hematologic cancers.

**Table 4:** Standardized incidence ratios (SIRs) for types of cancer in patients with SS Exp: expected; Obs: observed; CI: confidence interval; NHL: non-Hodgkin lymphoma; SS: Sjögren’s syndrome *Incidence of disease in 100,000 patient-years **Calculated using data from GLOBOCAN [11] for +25-year-olds

Categories	Total (n: 303)			
	Incidence in the general Turkish population*	Sjögren’s syndrome cohort		
		Obs (n)	Exp**	SIR (95% CI)
All cancers	415.7	27	12.59	2.25 (1.513-3.228)
Solid cancers	227.5	21	6.89	3.04 (1.937-4.58)
Thyroid	27.7	7	0.83	8.43 (3.689-16.68)
Breast	89.4	5	2.70	1.85 (0.687-4.105)
Hematological	27.4	6	0.83	7.22 (2.93-15.04)
NHL	11.4	4	0.34	11.76 (3.73-28.38)

For all cancers, the SIR was 2.25 (95% CI 1.513-3.228), which was higher compared to the overall population. For solid cancers and hematologic cancers, the SIR was 3.04 (95% CI 1.937-4.58) and 7.22 (95% CI 2.93-15.04), respectively. Among cancer subtypes, the SIR was 8.43 (95% CI 3.689-16.68) in thyroid cancer and 1.85 (95% CI 0.687-4.105) in breast cancer. Among hematologic cancers, the SIR in NHL was 11.76 (95% CI 3.73-28.38), which was higher than that of the overall population.

On logistic regression analysis, the presence of lymphopenia (p = 0.038) was revealed to be an independent risk factor for the development of malignancy (Table 5).

**Table 5:** Binary logistic regression analysis of the risk factors for cancers csDMARD: conventional synthetic disease-modifying antirheumatic drugs; anti-TNF: anti-tumor necrosis factor

	B	t	95% confidence interval		p
Gender	20.067	-	-	-	0.988
Age	0.041	1.042	0.998	1.088	0.064
Disease duration	0.011	1.011	0.887	1.152	0.871
Antinuclear antibody (+)	-2.304	0.100	-	-	0.996
Rheumatoid factor (+)	0.643	1.902	0.567	6.385	0.298
Anti-Ro (SSA) (+)	-0.398	0.672	0.228	1.984	0.471
Anemia	-0.730	0.482	0.159	1.461	0.197
Leukopenia	-0.305	0.737	0.241	2.254	0.593
Thrombocytopenia	-0.840	0.432	0.019	9.651	0.596
Lymphopenia	1.887	6.602	1.106	39.416	0.038
Use of corticosteroid	0.501	1.651	0.505	5.398	0.407
Use of hydroxychloroquine	1.232	3.427	0.689	17.042	0.132
Use of immunosuppressive	2.144	8.537	0.277	263.525	0.220
Use of csDMARD	-1.994	0.136	0.005	3.705	0.237
Use of anti-TNF	17.356	-	-	-	0.999

## Discussion

This study is one of the few that examines the elevated risk of cancer in Turkish patients with both pSS and sSS. It aimed to ascertain the elevated risk of both solid and hematological cancers and to identify the factors that influence this risk.

In the study, the incidence of both solid and hematologic malignancies increased in comparison to the regular population, which was similar in terms of age and gender. The calculated overall risk for cancer increased 2.24 times (SIR 2.25, 95% CI 1.513-3.228) (SIR 2.25, 95% CI 1.513-3.228). It is widely recognized that the incidence of NHL, especially hematologic malignancies, increases in pSS patients. However, different cancer types have been demonstrated to increase in solid malignancies in different studies. A 3.04-fold increase in the risk of solid cancer was observed in our study (SIR 3.04, 95% CI 1.937-4.58). This outcome corroborates comparable investigations [12,13].

In a large patient population, Liang et al. reported that NHL among hematologic malignancies and thyroid carcinoma among solid malignancies were associated with an increased risk in pSS patients. A statistically increased risk was observed in thyroid cancer, and NHL was observed in our study when compared to the overall population. In the same meta-analysis, the cancer rate was substantially higher in women, which is consistent with our study [8]. In a recent meta-analysis that was comparable to our study, an increase in the incidence of NHL and thyroid cancer was observed [14]. The frequency of NHL, breast, and thyroid malignancies among solid organs has increased in the database submitted from various countries [15].

In the Mendelian randomization analysis study examining the relationship between SS and cancers conducted by Jia and colleagues, it was suggested that lymphoma risk increased, the risk of prostate and endometrial cancer diminished, and there was a causal relationship with liver, biliary, and urinary tract cancers. Significant associations with thyroid, breast, and lung cancers were not identified. The results indicate that there is no causal relationship between SS and breast cancer subtypes [16].

Several epidemiologic studies have demonstrated that autoimmune diseases elevate the risk of cancer development. In SS, B-cell abnormality and chronic inflammation exacerbate this risk [17]. In patients with SS, the development of lymphoma is predicted by cryoglobulin-related markers, lymphoid infiltration of the salivary gland, EULAR SS disease activity index, and other factors [18].

Another review has shown that the development of lymphoma in pSS is significantly influenced by the chronic activation of lymphocytes, cytokines, B-cell activating factor (BAFF), and NfkB, as well as lymphocyte infiltration into target tissues [19].

Some developmental scores for lymphoma have been established, and they include salivary gland enlargement, RF positivity, lymphadenopathy, monoclonal gammopathy, Raynaud’s phenomenon, anti-Ro/SSA and/or anti-La/SSB autoantibodies, and low C4. A score of ≤2 suggests a low risk of lymphoma, while an increase in the score augments the risk of lymphoma [19-21].

Studies have demonstrated that geoepidemiologic factors regulate the severity and type of organs and systems affected in pSS. Age and gender have also been reported to influence the pattern of involvement and prognosis. However, research on the factors associated with the development of malignancy is scarce [6,22]. We intended to evaluate these factors in this investigation.

In the study conducted by Kang et al., pSS patients over the age of 50 were found to have an increased incidence of thyroid cancer in women and lung cancer in men, in addition to an increase in the incidence of NHL. In the same study, there was also an increase in the incidence of oropharyngeal cancer [23]. Additionally, this study emphasizes the association between age and sex. In our study, no significant difference was observed in cancer incidence based on age, disease duration, and sex. However, lymphopenia was found to increase the risk of cancer. Logistic regression analysis showed a sixfold increase in risk. The cohort was composed of 95% female patients, and all the patients who developed malignancy were women. Therefore, no statistically significant difference was observed. However, studies also revealed that malignancy was more prevalent in women [24,25].

Since the incidence of colonic adenoma could not be determined through GLOBOCAN, corresponding statistics could not be calculated. However, colonic adenomas were detected in pSS patients in certain investigations. Additional studies are needed to ascertain the risk. In our study, we identified tubular adenoma in the colon in five patients [26].

This study has several limitations. As a retrospective, single-center study, the findings may not be generalizable to larger populations. The small sample size and the predominance of female patients (95%) limited the evaluation of sex-related differences in cancer risk. Body mass index (BMI), comorbidities, family history of malignancy, and severity of Sjögren's syndrome, which may affect the interpretation of cancer risk, could not be assessed. The absence of longitudinal follow-up may have led to underreporting of cancers diagnosed after the study period. Future prospective, multicenter studies including comprehensive risk factors and disease severity are needed to confirm these findings and further clarify malignancy risk in SS patients. The number of studies that will allow geoepidemiological comparisons in the Turkish population is quite limited. Although this study is an important step toward filling the gap in this field, it needs to be supported with larger sample groups.

## Conclusions

In conclusion, this study provides a comprehensive assessment of the development of solid and hematologic malignancies in the SS cohort. The prevalence of malignancies was found to be high at 7.6%, with an overall cancer risk increased by 3.04-fold. Among solid malignancies, thyroid cancer (2.3%) was the most common type, while NHL (1%) was the most frequently observed hematologic malignancy. In SS patients, the risk of thyroid cancer was significantly elevated, with an overall cancer risk increased by 8.43-fold. Consistent with expectations, a significant increase in NHL risk was also observed, with an 11.76-fold higher risk. In this context, regular screening for NHL and thyroid cancer in pSS patients with risk factors is of critical clinical importance.
